# Clinicopathological and immunohistochemical characteristics of bullous pilomatricoma: a retrospective, single-center study, and comparison with ordinary pilomatricoma^☆^

**DOI:** 10.1016/j.abd.2023.06.007

**Published:** 2024-02-12

**Authors:** Kyung-Hwa Nam, Sang-Kyung Lee, Il-Jae Lee, Jin Park, Seok-Kweon Yun

**Affiliations:** a Department of Dermatology, Jeonbuk National University Medical School, Jeonju, South Korea; b Research Institute of Clinical Medicine of Jeonbuk National University – Biomedical Research Institute of Jeonbuk National University Hospital, Jeonju, South Korea

**Keywords:** Immunohistochemistry, Matrix metalloproteinases, Pathology, clinical, Pilomatrixoma, Vascular endothelial growth factors

## Abstract

**Background:**

Bullous pilomatricoma is a rare variant of pilomatricoma. As it has been published in sporadic case reports, a limited understanding of its clinicopathological characteristics restricts its effective diagnosis and treatment.

**Objectives:**

This study aimed to analyze the clinicopathological and immunohistochemical characteristics of bullous pilomatricoma to better understand the bullous transformation of pilomatricoma.

**Methods:**

The authors conducted a retrospective study of 12 patients with bullous pilomatricoma and compared their clinical, histopathological, and immunohistochemical data with those of patients with ordinary pilomatricoma.

**Results:**

Bullous pilomatricoma showed no sex preference, with a mean onset age of 31.2 years. The common sites were the upper extremities and trunk. Bullous pilomatricoma had a shorter disease duration, a larger diameter, and a greater tendency to increase in size than those of ordinary pilomatricoma. Histopathologically, bullous pilomatricoma had a shorter duration, lesser calcification, more mitotic figures, and distinct dermal features from those of ordinary pilomatricoma. Immunohistochemically, the expression of Matrix Metalloprotease (MMP)-2, MMP-9, vascular endothelial growth factor receptor-3 (VEGFR-3), and VEGF-C was elevated.

**Study limitations:**

The study was retrospective, and the sample size was small.

**Conclusion:**

The distinctive features of bullous pilomatricoma potentially result from dermal changes associated with the release of angiogenic factors and proteolytic enzymes. This comprehensive analysis provides novel insights into the clinical features and pathogenesis of bullous pilomatricoma.

## Introduction

Pilomatricoma, also known as calcifying epithelioma of Malherbe, is a benign neoplasm originating from hair matrix cells.^1^, ^2^, ^3^ It presents as a solitary, firm, deep-seated nodule with normal overlying skin. Bullous pilomatricoma is a rarely reported morphological variant, characterized by a soft, heavily folded, striae-like skin appearance overlying the tumor or pseudo-bulla formation.^4^, ^5^, ^6^, ^7^, ^8^, ^9^ Histopathological features of disrupted dermal collagen fibers, dermal lymphatic dilation, and lymphedema have been reported in cases of bullous pilomatricoma.^9^, ^10^ To date, only a few sporadic cases of bullous pilomatricoma have been published as case reports, with one literature review.^5^, ^6^, ^7^, ^8^, ^9^, ^11^, ^12^, ^13^, ^14^, ^15^, ^16^, ^17^, ^18^, ^19^ A Limited understanding of clinicopathological characteristics hampers proper clinical diagnosis and treatment.

Various theories have been proposed to explain the origin of bullous type pilomatricoma. 1) Mechanical irritation, 2) Obstruction of lymphatic vessels and congestion of lymphatic fluid caused by the pressure of the pilomatricoma’s hardcore, and 3) The release of elastolytic enzymes from tumor cells or infiltrating inflammatory cells to induce disruption of collagen fibers and dilatation of lymphatics have been implicated as triggering factors.^7^, ^8^, ^9^ In this study, the authors aimed to identify new informative features, including traumatic history, tumor nodule size, disease duration, and angiogenic/lymphangiogenic factors or proteolytic enzymes with bullous pilomatricoma.

The authors evaluated the clinicopathological and immunohistochemical features of 12 patients with bullous pilomatricoma and assessed the development of bullous morphology. This is the first comprehensive study to analyze bullous pilomatricoma cases diagnosed in a single center in Korea.

## Methods

### Patients

The authors retrospectively studied 12 patients diagnosed with bullous pilomatricoma at the JBNUH between January 2009 and May 2018. This study was approved by the institutional review board. Informed consent was obtained from all patients.

### Clinical findings

The following demographic and clinical data were collected: age, sex, site, duration, clinical impression, history of trauma, size, and change in size.

### Histopathological findings

The biopsy samples were fixed in 10% formalin and embedded in paraffin wax. Then, 4 µm-thick sections were stained with Hematoxylin and Eosin (H&E), elastic (Roche, Basel, Switzerland), and Elastica-van Gieson (EVG, Muto, Tokyo, Japan). Two dermatopathologists evaluated the following histopathological data from Hematoxylin and Eosin (H&E) staining of all biopsied specimens: tumor (chronological stage, squamous epithelium, basaloid epithelium, transitional cell, shadow cell, inflammatory infiltrates, giant cell, calcification, and mitosis) and dermal changes (dilatation of lymphatics and blood vessels, increase in the number of lymphatics and blood vessels, edema, increase in the interstitial space, sparsity and disruption of collagen fibers, and sparsity of elastic fibers). The chronological stages were histopathologically categorized into early, fully developed, early regressive, and late regressive.^20^ The early lesion (Stage 1) was a small cyst lined by squamous and basaloid epithelium containing keratin filaments and shadow cells. The fully developed lesion (Stage 2) was a large cornified eosinophilic mass containing shadow cells with a peripheral lining of the basaloid epithelium. Early regressive lesions (Stage 3) showed inflammatory infiltrates with multinucleated histiocytic giant cells. At the periphery, small foci of basaloid cells were found. The late regressive lesion (Stage 4) was an irregularly shaped, confluent mass of eosinophilic hair matrix material and shadow cells, with varying degrees of calcification and ossification. It was surrounded by hyalinized and sclerotic stroma. This stage had very few, if any, inflammatory infiltrates. When two or more stages were observed simultaneously, the stage with the most dominant features was selected. The determination of the dermal histopathological findings of collagen and elastic fibers was re-examined by Elastic and EVG staining of all the specimens.

### Immunohistochemical findings

Immunohistochemical staining was performed on paraffin-embedded sections, according to the manufacturer’s instructions and established laboratory protocols. The following primary antibodies were used: CD31 (Dako, Glostrup, Denmark; dilution 1:1000), CD34 (Roche, Basel, Switzerland; RTU), D2-40 (Roche, Basel, Switzerland; RTU), MMP-2 (Abcam, Cambridge, UK; ab97779; dilution 1:1000), MMP-9 (Abcam, Cambridge, UK; ab137867; dilution 1:2000), VEGFR-3 (Abcam, Cambridge, UK; ab27278; dilution 1:100), and VEGF-C (Abcam, Cambridge, UK; ab9546; dilution 1:100). The dermal histopathological findings of lymphatic and blood vasculature were re-examined for all specimens using CD31, CD34, and D2-40 staining by two dermatopathologists. Two dermatopathologists also evaluated the immunoreactivity of MMP-2, MMP-9, VEGFR-3, and VEGF-C using the H-score, which assesses both the staining intensity and fraction of stained cells at each intensity.^21^, ^22^ The staining intensity was scored as “0” for no evidence of staining, “1+” for weak staining, visible with high magnification, “2+” for moderate staining, “3+” for strong staining, and visible at low magnification. The percentage of stained cells (0%–100%) at each staining intensity was determined visually. The H-score was calculated by the formula: 0×(percentages of “0” cells) + 1×(percentages of “1+” cells) + 2×(percentages of “2+” cells) + 3×(percentages of “3+” cells), with a range of 0 to 300.

### Comparative analysis of bullous and ordinary pilomatricoma

To comprehensively investigate the differences in clinical features between bullous and ordinary pilomatricoma, the authors analyzed 159 cases from 150 patients with biopsy-proven ordinary pilomatricoma during the same period at JBNUH. Five patients had two lesions; one patient had three pilomatricoma-related lesions, and two patients experienced recurrence. To analyze the histopathological and immunohistopathological features of the dermis and tumor, 143 cases of ordinary pilomatricoma specimens were excluded: 1) Enucleation biopsied tumor specimen without dermal tissue or 2) Torn, disoriented specimen because it was not sufficient to evaluate the features of bullous transformation in the dermis. The histopathological and immunohistopathological features of 16 completely excised ordinary pilomatricoma specimens, including the epidermis, dermis, and tumor, were evaluated.

### Statistical analysis

Statistical analyses were performed using SPSS version 25 (SPSS Inc., Chicago, IL, USA) and R version 4.2.1. All clinical and histopathological data were analyzed using the chi-square test or Fisher’s exact test, except for the chronological stage data. The authors used the chi-square test if the expected cell frequencies < 5 were less than 20%, and Fisher’s exact test if the expected cell frequencies < 5 were more than 20%. The chi-square test was used to analyze the data of the squamoid epithelium, increased lymphatics, dilated blood vessels, edema, increased interstitial space, sparse elastic fibers, and sparse collagen fibers, whereas the other data were analyzed using Fisher’s exact test. Meanwhile, the chronological stage (Stages: 1–4) and immunohistochemical reactivity (H-score: 0–300) were evaluated using R, the Wilcoxon rank sum test because their ordered categories exhibited a significant linear trend. Results with a p-value < 0.05 were considered statistically significant.

## Results

### Clinical findings

Bullous pilomatricoma showed no gender preference, and ordinary pilomatricoma had a female predilection (M:F ratio = 1:1.79). Bullous pilomatricoma occurred predominantly in patients aged 10–19 years (58.3%), followed by those aged 50–59 years (25.0%). Conversely, ordinary pilomatricoma occurred predominantly in patients aged 0–9 years (44.7%), followed by those aged 10–19 years (31.4%). The mean ages of onset were 31.2 and 15.5 years for bullous pilomatricoma and ordinary pilomatricoma, respectively (Table 1).

**Table 1:** Demographic data of patients with pilomatricoma.

	Bullous pilomatricoma		Ordinary pilomatricoma		p-value
**Number of patients**	12		150		
**Number of cases**	12		159^b^		
**Sex**					0.362
**Male**	6/12	(50.0%)	57/159	(35.8%)	
**Female**	6/12	(50.0%)	102/159	(64.2%)	
**Sex ratio (M:F)**	1:1		1:1.79		
**Age (years)**					0.001^a^
** 0–9**	0/12	(0%)	71/159	(44.7%)	
** 10–19**	7/12	(58.3%)	50/159	(31.4%)	
** 20–29**	1/12	(8.3%)	12/159	(7.5%)	
** 30–39**	0/12	(0%)	11/159	(6.9%)	
** 40–49**	0/12	(0%)	7/159	(4.4%)	
** 50–59**	3/12	(25.0%)	6/159	(3.8%)	
** 60–69**	0/12	(0%)	1/159	(0.6%)	
** 70–79**	0/12	(0%)	0/159	(0%)	
** 80–89**	1/12	(8.3%)	1/159	(0.6%)	
** Mean age**	31.2	years	15.5	years	

a p-values of <0.05 were considered statistically significant.

b Among 150 patients with ordinary pilomatricoma, five patients had two lesions, one patient had three pilomatricoma-related lesions, and two patients experienced recurrence.

Only 5 (41.7%) bullous pilomatricoma cases were clinically diagnosed as pilomatricoma, while 66% of ordinary pilomatricoma cases were clinically diagnosed as pilomatricoma. Bullous pilomatricoma was found most frequently in the upper extremities (41.7%), followed by the trunk (25.0%), while ordinary pilomatricoma was found most frequently in the face (54.1%), followed by the upper extremities (22.6%).

**Figure 1:**
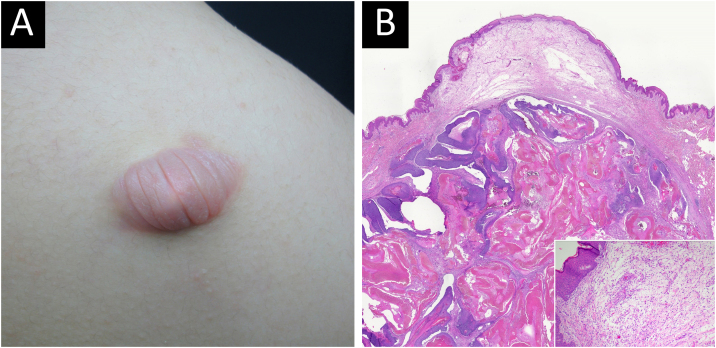
Bullous pilomatricoma. Clinical appearance (A) and histopathological features (B) Chronological stage 2, Hematoxylin & Eosin, scanning view, inset: ×100.

Bullous pilomatricoma was detected in 10/11 (90.9%) patients within three months, and ordinary pilomatricoma was detected in only 43/134 (32.1%) patients within the same period. The size of bullous pilomatricoma was greater than 1.0 cm in all 12 patients (100%), whereas that of ordinary pilomatricoma was less than 1.0 cm in 73/159 (52.2%) patients. The mean tumor size was 1.68 cm for bullous pilomatricoma and 0.99 cm for ordinary pilomatricoma. All recorded cases of bullous pilomatricoma (8/8, 100%) complained of an increase in tumor size. Four patients with bullous pilomatricoma had a history of trauma to the lesion, and 18 patients with ordinary pilomatricoma had a history of injury (Table 2, Fig. 1).

**Table 2:** Clinical differences between bullous and ordinary pilomatricoma.

	Bullous pilomatricoma		Ordinary pilomatricoma		p-value
**Clinical impression**^a^					
** Pilomatricoma**	5/12	(41.7%)	105/159	(66.0%)	<0.001^a^
** Hemangioma**	3/12	(25.0%)	0/159	(0.0%)	
** Epidermal cyst**	0/12	(0.0%)	37/159	(23.3%)	
** Trichilemmal cyst**	1/12	(8.3%)	2/159	(1.3%)	
** Dermoid cyst**	0/12	(8.3%)	2/159	(1.3%)	
** Lipoma**	1/12	(0.0%)	2/159	(1.3%)	
** Others**	2/12	(16.7%)	11/159	(6.9%)	
** Total**	12/12	(100.0%)	159/159	(100.0%)	
**Location**^a^					
** Scalp**	2/12	(16.7%)	5/159	(3.1%)	0.002^a^
** Face**	1/12	(8.3%)	86/159	(54.1%)	
** Neck**	1/12	(8.3%)	18/159	(11.3%)	
** Upper extremity**	5/12	(41.7%)	36/159	(22.6%)	
** Lower extremity**	0/12	(0%)	4/159	(2.5%)	
** Trunk**	3/12	(25.0%)	10/159	(6.3%)	
** Total**	12/12	(100.0%)	159/159	(100.0%)	
**Duration**^a^					
** 0 < D ≤3 months**	10/11	(90.9%)	43/134	(32.1%)	0.001^a^
** 3 < D ≤12 months**	1/11	(9.1%)	65/134	(48.5%)	
** D >12 months**	0	(0.0%)	26/134	(19.4%)	
** Total**	11/11	(100.0%)	134/134	(100.0%)	
**NA**	1/12		25/159		
**Size**^a^					
**0 < S <0.5 cm**	0/12	(0%)	14/159	(8.8%)	<0.001^a^
**0.5 cm ≤ S <1.0 cm**	0/12	(0%)	69/159	(43.4%)	
** 1.0 cm ≤ S <1.5 cm**	5/12	(41.7%)	53/159	(33.3%)	
** 1.5 cm ≤ S <2.0 cm**	3/12	(25.0%)	16/159	(10.1%)	
** 2.0 cm ≤ S <2.5 cm**	2/12	(16.7%)	4/159	(2.5%)	
** 2.5 cm ≤ S <3.0 cm**	1/12	(8.3%)	1/159	(0.6%)	
** S ≥3.0 cm**	1/12	(8.3%)	2/159	(1.3%)	
** Total**	12/12	(100.0%)	159/159	(100%)	
**Change of size**					
** No change**	0/8	(0.0%)	23/90	(25.6%)	0.192
** Increase in size**	8/8	(100.0%)	64/90	(71.1%)	
** Decrease in size**	0/8	(0.0%)	3/90	(3.3%)	
** Total**	8/8	(100.0%)	90/90	(100.0%)	
** NA**	4/12		69/159		
**Trauma History**					
** History (+)**	4/6	(66.7%)	18/47	(38.3%)	0.219
** History (–)**	2/6	(33.3%)	29/47	(61.7%)	
** NA**	6/12		112/159		

NA, Not Available (complete information was not available from the medical records of patients); M, Male; F, Female; D, Duration; S, Size.

a p-values of <0.05 were considered statistically significant.

### Histopathological findings

In histopathologic data of tumors, the difference in chronological stage, calcification, and mitotic figures between patients with both types of pilomatricoma was statistically significant. Of the 12 patients with bullous pilomatricoma, 7 (58.3%) and 5 (41.7%) were diagnosed with chronological stages 3 and 2, respectively. Of the 16 patients with ordinary pilomatricoma, 12 (75.0%), 3 (18.8%), and 1 (6.3%) were diagnosed with stages 3, 4 and 2, respectively. Calcification and mitotic figures were observed in 5 (41.7%) and 8 (66.7%) patients with bullous pilomatricoma, respectively. Meanwhile, 14 (87.5%) and 3 (18.8%) patients with ordinary pilomatricoma showed calcification and mitotic figures, respectively.

Unlike ordinary pilomatricoma, nearly all patients with bullous pilomatricoma showed dermal changes, including dilated (66.7%) or increased (100%) lymphatics, dilated (83.3%) or increased (100%) blood vessels, dermal edema (100%), increased interstitial space (100%), sparse elastic fibers (100%), sparsity (100%), and disruption (66.7%) of collagen fibers (Table 3, Fig. 1).

**Table 3:** Histopathological differences between bullous and ordinary pilomatricoma.

	Bullous Pilomatricoma		Ordinary Pilomatricoma		p-value
**Tumor**					
** Chronological stage**^a^					0.013^a^
** 1. Early**	0/12	(0.0%)	0/16	(0.0%)	
** 2. Fully developed**	5/12	(41.7%)	1/16	(6.3%)	
** 3. Early regressive**	7/12	(58.3%)	12/16	(75.0%)	
** 4. Late regressive**	0/12	(0.0%)	3/16	(18.8%)	
** Squamoid epithelium**	5/12	(41.7%)	7/16	(43.8%)	0.912
** Basaloid epithelium**	12/12	(100.0%)	13/16	(81.3%)	0.238
** Transitional cell**	12/12	(100.0%)	11/16	(68.8%)	0.053
** Shadow cell**	12/12	(100.0%)	16/16	(100.0%)	
** Inflammatory infiltrates**	12/12	(100.0%)	15/16	(93.8%)	1
** Giant cell**	12/12	(100.0%)	14/16	(87.5%)	0.492
** Calcification**^a^	5/12	(41.7%)	14/16	(87.5%)	0.017^a^
** Mitosis**^a^	8/12	(66.7%)	3/16	(18.8%)	0.019^a^
**Dermal change**					
** Dilated lymphatics**^a^	8/12	(66.7%)	2/16	(12.5%)	0.005^a^
** Increased lymphatics**^a^	12/12	(100.0%)	1/16	(6.3%)	0.000^a^
** Dilated blood vessels**^a^	10/12	(83.3%)	4/16	(25.0%)	0.006^a^
** Increased blood vessels**^a^	12/12	(100.0%)	6/16	(37.5%)	0.001^a^
** Edema**^a^	12/12	(100.0%)	0/16	(0.0%)	<0.001^a^
** Increased interstitial space**^a^	12/12	(100.0%)	0/16	(0.0%)	<0.001^a^
** Sparse elastic fibers**^a^	12/12	(100.0%)	0/16	(0.0%)	<0.001^a^
** Sparse collagen fibers**^a^	12/12	(100.0%)	0/16	(0.0%)	<0.001^a^
** Disruption of collagen fibers**^a^	8/12	(66.7%)	0/16	(0.0%)	<0.001^a^

a The p-values of < 0.05 were considered statistically significant.

### Immunohistochemical findings

**Figure 2:**
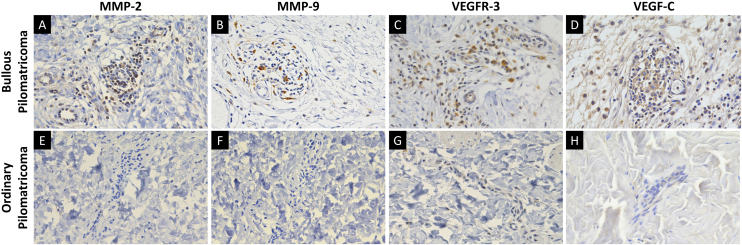
Immunohistochemical staining for MMP-2, MMP-9, VEGFR-3, and VEGF-C in bullous pilomatricoma (A–D) and ordinary pilomatricoma (E–H) with magnification (×400).

**Figure 3:**
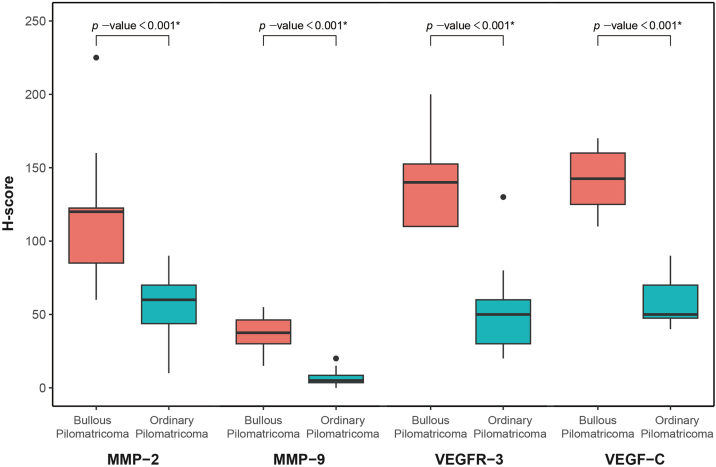
Box plot showing the result of immunohistochemical analysis using H-score in bullous and ordinary pilomatricoma for MMP-2, MMP-9, VEGFR-3, VEGF-C by Wilcoxon rank sum test.

MMP-2, MMP-9, VEGFR-3, and VEGF-C were expressed in dermal inflammatory cells and endothelial cells (Fig. 2). In patients with bullous pilomatricoma, MMP-2- and MMP-9-positive cells had higher H-scores than those in patients with ordinary pilomatricoma. Moreover, VEGFR-3 and VEGF-C expression levels in patients with bullous pilomatricoma were higher than those in patients with ordinary pilomatricoma. The differences in immunoreactivity of MMP-2, MMP-9, VEGFR-3, and VEGF-C in the bullous and ordinary groups were statistically significant (Fig. 3).

## Discussion

Bullous pilomatricoma is a rare variant of pilomatricoma that usually presents as a semi-transparent, wrinkled blister with a firm underlying nodule.^9^ In the present study, three-fifths of the cases with bullous pilomatricoma were clinically misdiagnosed as other diseases before histopathological confirmation, highlighting that the peculiar morphology of bullous pilomatricoma makes diagnosis difficult. Nonetheless, the present results revealed that bullous pilomatricoma has distinguishing demographic and clinical features in terms of age of onset, predilection sites, tumor size, and growth rate, which could facilitate a proper diagnosis. Approximately 60% of pilomatricoma develop in the first two decades of life, mostly in the first decade,^1^, ^4^, ^23^ and the ordinary group in this study showed a similar age distribution. Bullous pilomatricoma in this study did not affect individuals under the age of ten, and the mean age of patients susceptible to bullous pilomatricoma was higher than that of patients susceptible to ordinary pilomatricoma (31.2 years vs. 15.5 years). The common sites of pilomatricoma lesions are typically the head, upper limbs, neck, trunk, and lower limbs, in decreasing order of frequency,^1^, ^2^, ^24^ as shown in patients with ordinary pilomatricoma in this study. Bullous pilomatricoma appeared most frequently on the upper extremities (n=5, 41.7%) and trunk, especially the shoulder (n=3, 25.5%). These results are consistent with the review by Chen et al., in which 11/16 reported cases of bullous pilomatricoma were found on the shoulder or upper arm.^9^ In this study, bullous pilomatricoma clinically showed shorter disease duration, larger tumor size, and an increase in tumor size compared with ordinary pilomatricoma. Bullous pilomatricoma histopathologically revealed an earlier chronological stage, less calcification, and more mitotic figures than ordinary pilomatricoma. These results suggest that bullous pilomatricoma increases by over 1 cm in size in a relatively short period. Accordingly, early diagnosis and surgical decisions are crucial for the management of bullous pilomatricoma.

Bullous pilomatricoma has been designated as pseudobullous pilomatricoma in some literature because the blister-like spaces are filled with lymphatic fluid.^6^ In this study, bullous pilomatricoma showed degradation of connective tissue and development of vasculature in the dermis, whereas ordinary pilomatricoma showed only minimal histopathological changes in the dermal vasculatures. These distinct dermal histopathological features were consistent with its bullous morphology which is, more precisely, pseudobullous changes. Members of the MMP family can proteolytically degrade the extracellular matrix and facilitate cell migration.^25^ Several studies have demonstrated that MMPs are involved in the development of mid-dermal elastolysis and neoangiogenesis.^26^, ^27^, ^28^ A recent study demonstrated an association between MMP-9 and-12 expression and three cases of bullous-like/anetodermic pilomatricoma.^19^ Hence, the authors evaluated the expression of MMP-2 and MMP-9, members of the gelatinase family, which were found to be highly expressed in the dermal inflammatory cells of bullous pilomatricoma. It is assumed that these proteins facilitate the degradation of collagen and elastin fibers and, hence, the tumor growth of bullous pilomatricoma.

The primary role of the lymphatic system is to maintain tissue fluid homeostasis, the dysfunction of which can result in lymphedema and pathological lymphangiogenesis, causing inflammation and tumor metastasis.^29^, ^30^ VEGFR-3 and its ligand, VEGF-C, are potent inducers of lymphatic vascular development.^29^ This study showed that VEGFR-3 and VEGF-C expression was significantly higher in dermal inflammatory cells and vascular endothelium with bullous pilomatricoma than in those with ordinary pilomatricoma. This is the first study on the expression of VEGF-C and VEGFR-3 in bullous pilomatricoma. It is important to investigate further whether VEGF-C- and VEGFR-3-induced lymphangiogenesis in patients with bullous pilomatricoma is associated with a dermal inflammatory response and/or tumor progression.

Various theories have been proposed to explain the origin of bullous pilomatricoma. Inui et al. suggested that mechanical irritation can result in the formation of bullous pilomatricoma.^7^ However, in this study, only four patients with bullous pilomatricoma had a history of trauma, and none of the 18 patients with ordinary pilomatricoma who had a traumatic accident developed bullous tumors. According to Chen et al., a local mechanical scratching history was reported in only 2 out of 16 cases of bullous pilomatricoma.^9^ Meanwhile, the prevalence of bullous pilomatricoma in the upper extremities and arms implies a high probability of exposure to trauma, and patients might have forgotten the traumatic events with unrecognizable weak intensity, such as friction. Therefore, the theory proposing a mechanical irritation-induced bullous transformation of ordinary pilomatricoma requires further validation.

In addition, it has been suggested that additional pressure on the pilomatricoma’s hardcore or the growth area of the tumor nodule can block lymphatic vessels and cause lymphatic fluid leakage.^7^, ^8^ It has been further suggested that the tumor and/or inflammatory cells secrete elastolytic enzymes that degrade the extracellular matrix.^7^, ^9^ In this study, bullous changes were not observed in 26 patients with ordinary pilomatricoma that lasted more than 1 year and in 76 patients with ordinary pilomatricoma of size greater than 1 cm. Overall, bullous pilomatricoma may occur from the dermal release of MMPs, VEGFR-3, and VEGF-C rather than the degenerative change induced by traumatic stimulus or the growing or longstanding tumor nodule pressure, although additional studies are needed.

## Conclusion

This study revealed that bullous pilomatricoma is clinically characterized by a rapidly growing tumor greater than 1 cm with a predilection for the upper extremity and shoulder in older patients and histopathologically by loose dermal connective tissue with vasculature development, compared with ordinary pilomatricoma. These distinct clinicopathological features are likely to be associated with the dermal release of MMPs, VEGFR-3, and VEGFR-C. While further investigation with a larger cohort is warranted, the present study might improve the present understanding of the pathogenesis and aid in the diagnosis and treatment of bullous pilomatricoma.

## Financial support

None declared.

## IRB approval status

They are reviewed and approved by the Institutional Review Board of JBNUH (IRB nº 2019-04-061-001).

## Conflicts of interest

None declared.
